# Dysregulated expression of androgen metabolism genes and genetic analysis in hypospadias

**DOI:** 10.1002/mgg3.1346

**Published:** 2020-06-08

**Authors:** Zhongzhong Chen, Xiaoling Lin, Yaping Wang, Hua Xie, Fang Chen

**Affiliations:** ^1^ Department of Urology Shanghai Children’s Hospital Shanghai Jiao Tong University Shanghai China; ^2^ Department of Urology Huashan Hospital Fudan University Shanghai China; ^3^ Department of Urology Shanghai Jiao Tong University Affiliated Sixth People’s Hospital Shanghai China; ^4^ Shanghai Eastern Urological Reconstruction and Repair Institute Shanghai China

**Keywords:** *AR*, *CYP19A1*, hypospadias, gene expression, whole exome sequencing

## Abstract

**Background:**

The aberrant expression of genes involved in androgen metabolism and genetic contribution are unclear in hypospadias.

**Methods:**

We compared gene expression profiles by RNA sequencing from five non‐hypospadiac foreskins, five mild hypospadiac foreskins, and five severe hypospadiac foreskins. In addition, to identify rare coding variants with large effects on hypospadias risk, we carried out whole exome sequencing in three patients in a hypospadias family.

**Results:**

The average expression of androgen receptor (*AR*) and *CYP19A1* were significantly decreased in severe hypospadias (*p* < .01) and mild hypospadias (*p* < .05), whereas expression of several other androgen metabolism enzymes, including *CYP3A4*, *HSD17B14*, *HSD3B7*, *HSD17B7*, *CYP11A1* were exclusively significantly expressed in severe hypospadias (*p* < .05). Compound rare damaging mutants of *AR* gene with *HSD3B1* and *SLC25A5* genes were identified in the different severe hypospadias.

**Conclusions:**

In conclusion, our findings demonstrated that dysregulation of *AR* and *CYP19A1* could play a crucial role in the development of hypospadias. Inconsistent *AR* expression may be caused by the feedback loop of ESR1 signaling or combined genetic effects with other risk genes. This findings complement the possible role of *AR* triggered mechanism in the development of hypospadias.

## Graphical abstract

AR and CYP19A1 were significantly decreased in severe and mild hypospadias. AR and ER signaling could play a crucial role in the development of hypospadias. Inconsistent AR expression may be caused by the feedback loop of ER expression or combined genetic effects with other risk genes.

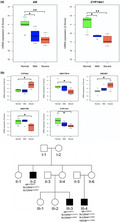

## 1 INTRODUCTION

Hypospadias (OMIM#: 300633) is among the most frequently congenital diseases in the male reproductive system. The average incidence of hypospadias is 2/1000 births, and the prevalence is suggested to be increasing during 1980–2010 (Yu et al., 2019). However, the exact biological mechanism underlying the development of hypospadias is unknown. Hypospadias is thought to be a multifactorial disorder, involving both genetic and environmental factors (Joodi et al., 2019). The hypospadias association studies demonstrated that a variety of genes and pathways are known to contribute the etiology of hypospadias, and these genes could affect the risk of hypospadias through protein‐protein interactions (PPIs) (Chen et al., 2019). Hormones are required for normal sex organs to grow into either male or female organs during the early development. Androgens and estrogens are the important steroid hormones responsible for the development of external genitalia (Balaji et al., 2020; Bouty, Ayers, Pask, Heloury, & Sinclair, 2015).

The penile development is controlled through the balance of androgen receptor (*AR*, OMIM#: 313700) to estrogen receptor α (*ESR1*, OMIM#: 133430) levels during the neonatal period (Zheng, Armfield, & Cohn, 2015). Oestrogenic agents and androgen‐blocking agents are the two major categories, which are used to induce hypospadias in rats and mice (Cunha, Sinclair, Risbridger, Hutson, & Baskin, 2015). Androgen signaling is regulated by the nuclear *AR* and play a pivotal role in masculinization during development. Disruption of androgen‐induced genes, such as *MAFB*, can interfere with urethral formation and results in hypospadias (Matsushita et al., 2018). Missense mutations of *AR* accounted for 3% of patients, which may play a critical role in the cause of isolated hypospadias (Kalfa et al., 2013). While any defect in the androgen signaling may lead to hypospadias, the *AR* expression in hypospadias are controversial with the variable results. When compared to the non‐hypospadiac children, *AR* expression exhibited various levels, including elevated (Balaji et al., 2020; Pichler et al., 2013), similar (Celayir, Moralioglu, Cetiner, Kir, & Celayir, 2019; Tack et al., 2019) or decreased (Lin, Xie, Chen, & Li, 2016; Silva et al., 2013). Various polymorphisms in *ESR1* are associated with the risk of hypospadias (Choudhry et al., 2015), and *ESR1* expression are also reported to be associated with hypospadias (Qiao et al., 2012; Wang et al., 2007). Despite the critical functions of androgen signaling and estrogen signaling in the process of hypospadias, regulation of gene expression and genetic contribution for hypospadias remain poorly unknown.

The exact molecular events for the development of hypospadias are just beginning to be addressed, the aim of this study was to investigate the gene expression profiles and the genetic basis to explore the possible biological mechanism, especially regulated by genes involved in androgen metabolism in hypospadias.

## 2 METHODS

### 2.1 Ethical compliance

The study was conducted after approval from the Ethics Committee of the Shanghai Children's Hospital in China (approve #: 2014R022‐F01 and 2020R018‐E01). Each patient was informed of the purpose of this study, and written consents were obtained from all participants or their parent/legal guardian. Ethical approval was obtained from the Shanghai Children's Hospital in China.

### 2.2 Human subjects

We performed RNA sequencing and exome sequencing study design in a male Han Chinese population. In all, 18 subjects, including 15 foreskin specimen for RNA sequencing and three blood samples for whole exome sequencing (WES) analysis, were included in this study.

For RNA sequencing analysis, 15 prepuces of children who underwent consecutive circumcision either because of phimosis (controls; *n* = 5) or because of hypospadias repair (mild hypospadias, *n* = 5; severe hypospadias; *n* = 5), were included in this study. Three hypospadias samples for WES were enrolled from a hypospadias family. The 18 samples were collected with mean age 4.4 ± 2.1 years during 2015 to 2018 at Shanghai Children's Hospital, Shanghai Jiaotong University. Patients with hypospadias were diagnosed by the Department of Urology.

### 2.3 RNA sequencing

The RNA was isolated from human tissues with TRIzol reagent according to the manufacturer's instructions (Life Technologies). RNA samples that pass quality parameters were used to construct RNA libraries using TruSeq RNA Library Prep Kit v2 (Illumina) according to the manufacturer protocol. The libraries were then sequenced on the Illumina NovaSeq 6000 platform with a paired‐end read length of 150 bp.

### 2.4 RNA analysis

RNA‐Seq fastq data was then carried out based on the following protocols. Skewer software (Jiang, Lei, Ding, & Zhu, 2014) was performed to dynamically remove the 3′ ends, linker sequences, and low mass fragments of raw reads. Quality assessment was examined by FastQC tool (www.bioinformatics.babraham.ac.uk/projects/fastqc/). Trimmed clean reads were aligned against human reference genome (hg38) by STAR (Dobin et al., 2013). Then, the transcriptome was assembled using StringTie (Pertea et al., 2015) software based on the Ensembl reference (Homo sapiens GRCh38.90) (Cunningham et al., 2019). The FPKM (Fragments Per Kilobase of transcript per Million mapped reads) method was applied to estimate differentially expressed genes.

The obtained RNA expression data was used for comparative analysis. We applied surrogate variable analysis (SVA) (Leek, Johnson, Parker, Jaffe, & Storey, 2012) to remove batch effects. The limma package (Smyth, 2004) was used to assess the differentially expressed genes between hypospadiac foreskins and non‐hypospadiac foreskins. The P‐values were adjusted for multiple comparisons using the False Discovery Rate (FDR). The adjusted *p* < .05 and 2‐fold were used as the cut‐off criteria for differentially expressed genes between severe hypospadias or hypospadias (including mild and severe hypospadias) and controls, whereas the *p* < .05 and 1.5‐fold were used as the cut‐off criteria for differentially expressed genes between mild hypospadias and controls. Function heatmap.2 was performed for the graphical display of the dendrogram (Gentleman et al., 2004). GeneSense (Chen et al., 2014) and STRING (https://www.string‐db.org) were used to identify PPIs encoded by hypospadias risk associated genes in human.

The principal component analysis (PCA) is performed to predict the relatedness of severe, mere and non‐hypospadiac samples based on the gene expression. 40 previous reported transcripts for enzymes (*AKR1C3*, *CYB5A*, *CYP11A1*, *CYP11B1*, *CYP11B2*, *CYP17A1*, *CYP19A1*, *CYP21A2*, *HSD17B1*, *HSD17B10*, *HSD17B11*, *HSD17B12*, *HSD17B13*, *HSD17B14*, *HSD17B2*, *HSD17B3*, *HSD17B4*, *HSD17B6*, *HSD17B7*, *HSD17B8*, *HSD3B1*, *HSD3B2*, *HSD3B7*, *RDH5*, *SRD5A1*, *SRD5A2*, *SRD5A3*, *STAR*, *AR*, *SHBG*, *AKR1C1*, *AKR1C2*, *AKR1C4*, *CYP3A4*, *CYP3A43, CYP3A5, CYP3A7, UGT2B15, UGT2B17* and *UGT2B7*) (Mitsiades et al., 2012) were analyzed to identify the dysregulated expressed genes participating in androgen metabolism in hypospadias. Two‐tailed Student's *t* tests were conducted to determine whether the androgen synthesis and metabolism related genes are differentially expressed between hypospadiac foreskins and non‐hypospadiac foreskins. All statistical analyses were performed by R software (http://www.R‐project.org).

### 2.5 WES, variant annotation and data analysis

We performed the WES analysis and variant annotation according to the method of Chen (Chen, Lei, Zheng, et al., 2018). As described in this method, coding variants were classified as synonymous, missense, LoF (loss of function, including splice acceptor/donor, stop gained/lost, initiator codon and frameshift indels) and others. The missense variants, that were predicted to be deleterious by SIFT (Kumar, Henikoff, & Ng, 2009) and damaging by PolyPhen‐2 (Adzhubei et al., 2010), were annotated as deleterious missense variants (D‐mis). Rare damaging variants (LoF and D‐mis) were selected that had a minor allele frequency (MAF) ≤1% in ExAC (http://exac.broadinstitute.org) and 1000 Genomes Project. Multiple alignments of the SLC25A5 proteins across species were performed by the CLUSTALW program built in Mega software (http://www.megasoftware.net/). RNA‐Seq data of 27 different tissues from 95 individuals, which is part of the Human Protein Atlas (www.proteinatlas.org) (BioProject: PRJEB4337) (Fagerberg et al., 2014), was downloaded from NCBI.

## 3 RESULTS

### 3.1 Molecular subtyping based on gene expression and hierarchical cluster analysis

**FIGURE 1:**
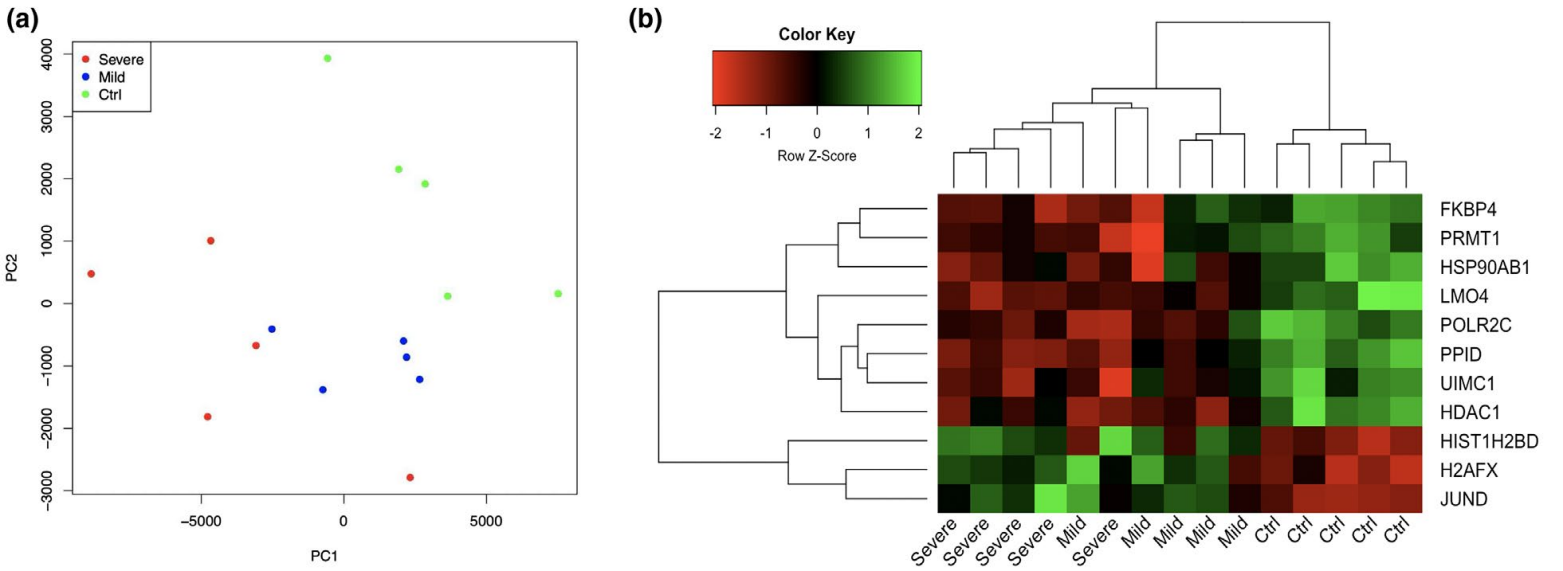
Comparison of gene expression in the severe and mild hypospadias. (a) The principal component analysis was performed using the first two principal components. Severe, severe hypospadiac foreskins; Mild, mild hypospadiac foreskins; Ctrl, non‐hypospadiac foreskins; (b) Hierarchical clustering of 11 differentially expressed genes, which encode proteins interacting with ESR1

Two different types (severe and mild) of hypospadiac foreskins and non‐hypospadiac foreskins (controls) were prepared in this study. Figure 1a showed that the gene expression patterns in severe hypospadias and controls are more different compared with mild hypospadias. Overall, 498 genes were identified differentially expressed in severe hypospadias, mild hypospadias and all (severe and mild) hypospadias compared with controls. Among these proteins encoded by differentially expressed genes, 11 proteins were predicted to interact with ESR1, while four proteins interact with AR. We performed unsupervised hierarchical clustering analysis of the 11 genes, which encode proteins interacting with ESR1. Results showed that the classifications of samples into hypospadias and controls (Figure 1b).

### 3.2 Analysis of differentially expressed genes in androgen synthesis and metabolism

**FIGURE 2:**
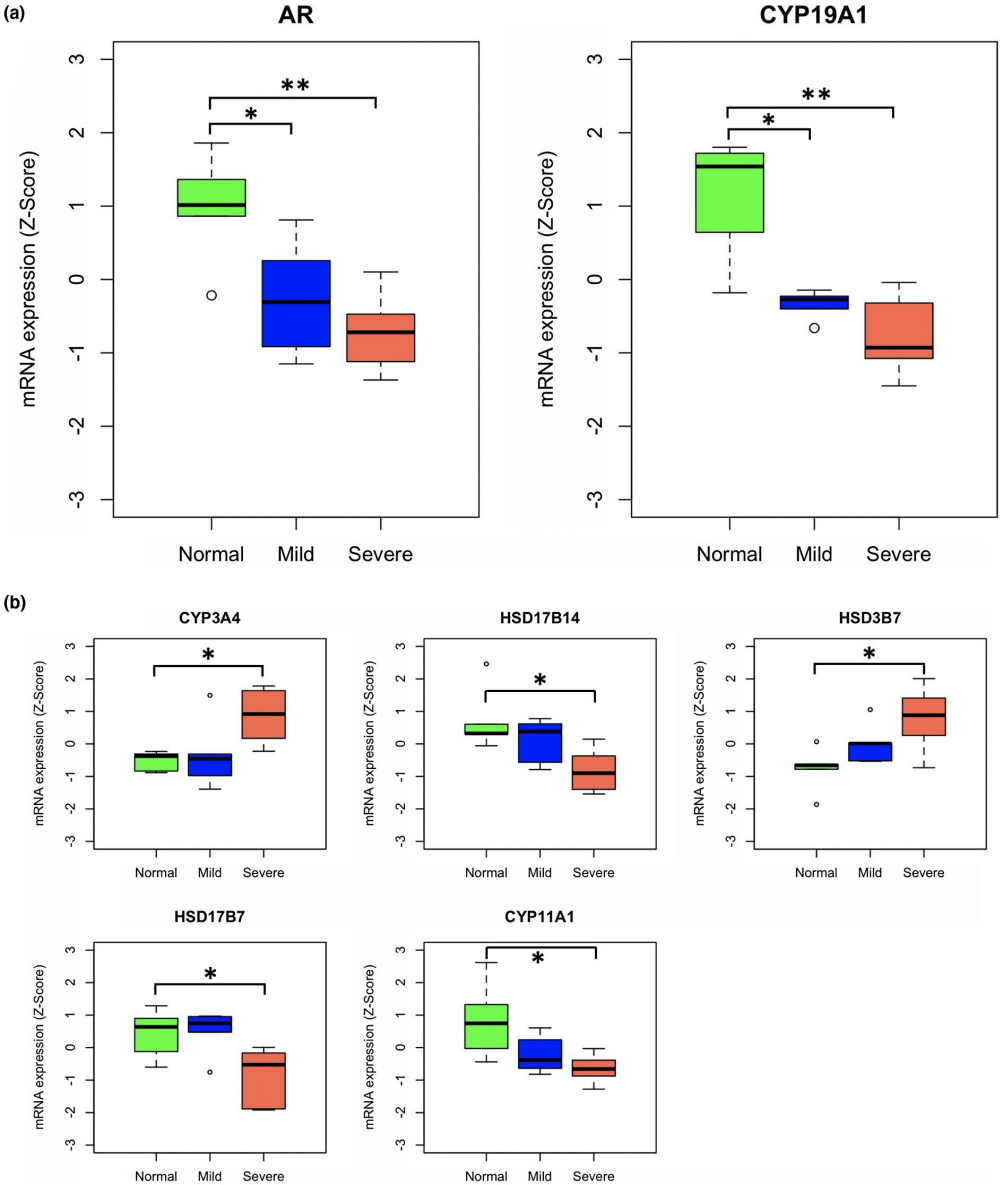
Boxplots of differentially mRNA expression for *AR*, *CYP19A1*, *CYP3A4, HSD17B14*, *HSD3B7*, *HSD17B7*, and *CYP11A1* in non‐hypospadiac tissue, mild and severe hypospadias. (a) *AR* and *CYP19A1* were significantly lower expressed in severe and mild hypospadias. (b) *CYP3A4, HSD17B14*, *HSD3B7*, *HSD17B7*, and *CYP11A1* were significantly expressed in severe hypospadias. The asterisk indicates a statistical difference (*p<.05, **p<.01) by Student's *t* test

Among 40 previous reported transcripts for enzymes involved in androgen synthesis and metabolism (Mitsiades et al., 2012), 36 (90%) genes were identified to be expressed (Table S1) and revealed high hypospadias variability. *AR* and *CYP19A1* (OMIM#: 107910) were found to be significantly decreased in severe hypospadias (*p* < .01) and mild hypospadias (*p* < .05) (boxplots for Z‐score mRNA expression are shown in Figure 2a). Importantly, compared to mild hypospadias, *AR* and *CYP19A1* showed lower expressed in severe hypospadias, whereas not reaching statistical significance in our panel. Moreover, *CYP3A4* (OMIM#: 107910), *HSD17B14* (OMIM#: 124010), *HSD3B7* (OMIM#: 607764), *HSD17B7* (OMIM#: 606756), and *CYP11A1* (OMIM#: 118485) were exclusively significantly expressed in severe hypospadias (*p* < .05) (boxplots for Z‐score mRNA expression are shown in Figure 2b).

### 3.3 Combined effects of *AR* variants and other risk variants cause diverse phenotypes in a three‐generation hypospadias family

**FIGURE 3:**
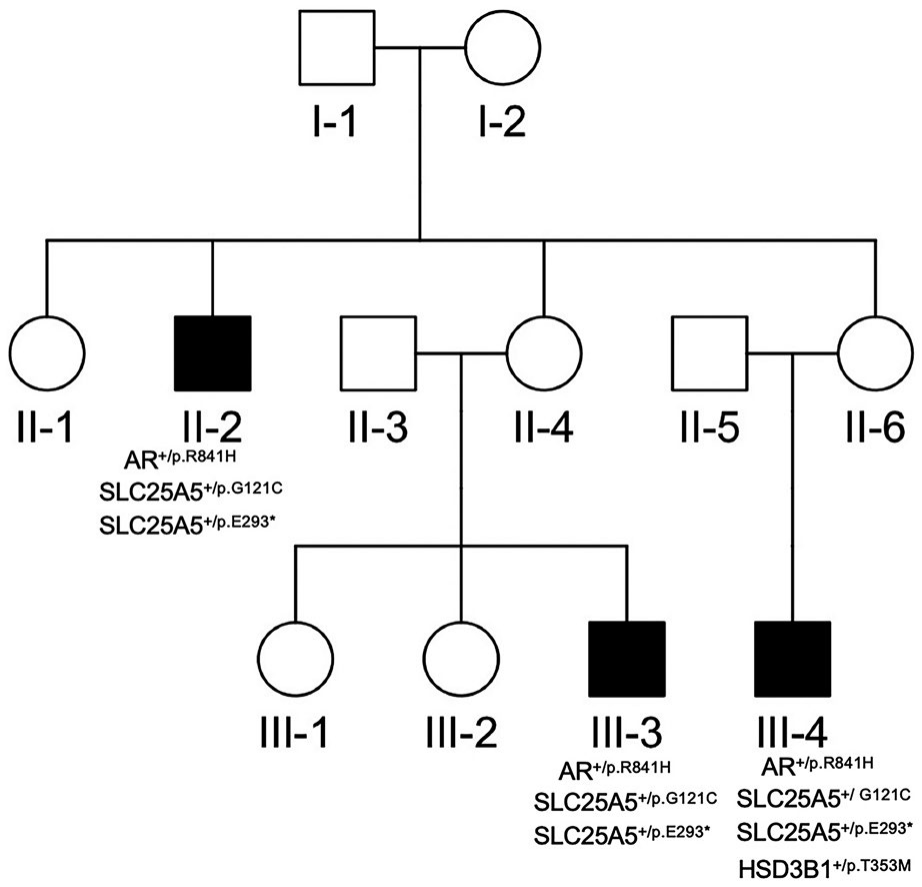
Pedigree analysis of a three‐generation family presenting with recessive inheritance of hypospadias. The pedigree plot with shapes for male (square), female (circle). The shapes are shaded and un‐shaded for hypospadias status of yes and no

The hypospadias family of this study included 12 individuals in a three‐generation pedigree (Figure 3) originating from Han Chinese. Six male family members were clinically investigated: all three cases showing severe hypospadias (II‐2, III‐3, III‐4) with the variable outcomes, and three individuals are unaffected. The urethral opening of these patients was located in the perineum. Among these three severe hypospadias, patient III3, III4 presented small penis with ventural curvature, bilateral accessible testis in scrotum. III‐4 was accompanied with crytorchidism and solitary kidney, and all of them have no other deformity.

The further WES data showed that the genetic interactions of rare damaging variants rather than single mutation yielded hypospadias with the variable outcomes. Firstly, we analyzed the rare damaging variants in 30 hypospadias risk associated genes (Chen et al., 2019), including *SHH*, *GLI1*, *GLI2*, *GLI3*, *FGF8*, *FGF10*, *FGFR2*, *BMP7*, *WT1*, *DGKK*, *HSD3B1*, *HSD17B3*, *SRD5A2*, *AR*, *ESR1*, *ESR2*, *BMP4*, *HOXA4*, *HOXB6*, *MAP3K1*, *CHD7*, *NR5A1*, *MAMLD1*, *HSD3B2*, *CYP11A1*, *AKR1C3*, *ATF3*, *BNC2*, *SP1*, *SP7*. Among all these detected rare variants in the 30 associated genes of the three hypospadias cases, four rare damaging variants were identified from three patients. Patients with rare damaging missense variants were observed to have different hypospadias phenotypes. The four mutants were distributed in three genes (Table 1). All of these damaging mutants are very rare in the Exome Aggregation Consortium (ExAC) database, with MAF < 0.01 (Table 1). The variant p.Arg841His or p.R841H (NM_000044.3: c.2522G>A) in *AR* gene were novel and absent in ExAC and 1000 Genomes Project (1KGP), while p.Thr353Met or p.T353M (NM_000862.2: c.1058C>T) in *HSD3B1* (OMIM#: 109715) gene did not exist in 1000 Genomes Project (Table 1).

**TABLE 1:** Rare damaging mutations were detected in *AR*, *SLC25A5* and *HSD3B1*

Sample	Gene	Nucleotide change	Amino acid change	Sex	SIFT^a^	PP2^b^	MAF in ExAC^c^
II−2	*AR*	c.2522G>A	p.Arg841His	M	D	P	NA^d^
	*SLC25A5*	c.877G>T	p.Glu293*	M	NA^d^	NA^d^	NA^d^
	*SLC25A5*	c.361G>T	p.Gly121Cys	M	D	D	5.5 × 10^−3^
III−3	*AR*	c.2522G>A	p.Arg841His	M	D	P	NA
	*SLC25A5*	c.877G>T	p.Glu293*	M	NA^d^	NA^d^	NA^d^
	*SLC25A5*	c.361G>T	p.Gly121Cys	M	D	D	5.5 × 10^−3^
III−4	*AR*	c.2522G>A	p.Arg841His	M	D	P	NA
	*SLC25A5*	c.877G>T	p.Glu293*	M	NA^d^	NA^d^	NA^d^
	*SLC25A5*	c.361G>T	p.Gly121Cys	M	D	D	5.5 × 10^−3^
	*HSD3B1*	c.1058C>T	p.Thr353Met	M	D	P	1.6 × 10^−5^

^a^ SIFT predictions: D, deleterious.

^b^ PolyPhen‐2 (PP2) predictions: B, benign; D, probably damaging; P, possibly damaging.

^c^ MAF from the Exome Aggregation Consortium (ExAC) database.

^d^ Not available.

**FIGURE 4:**
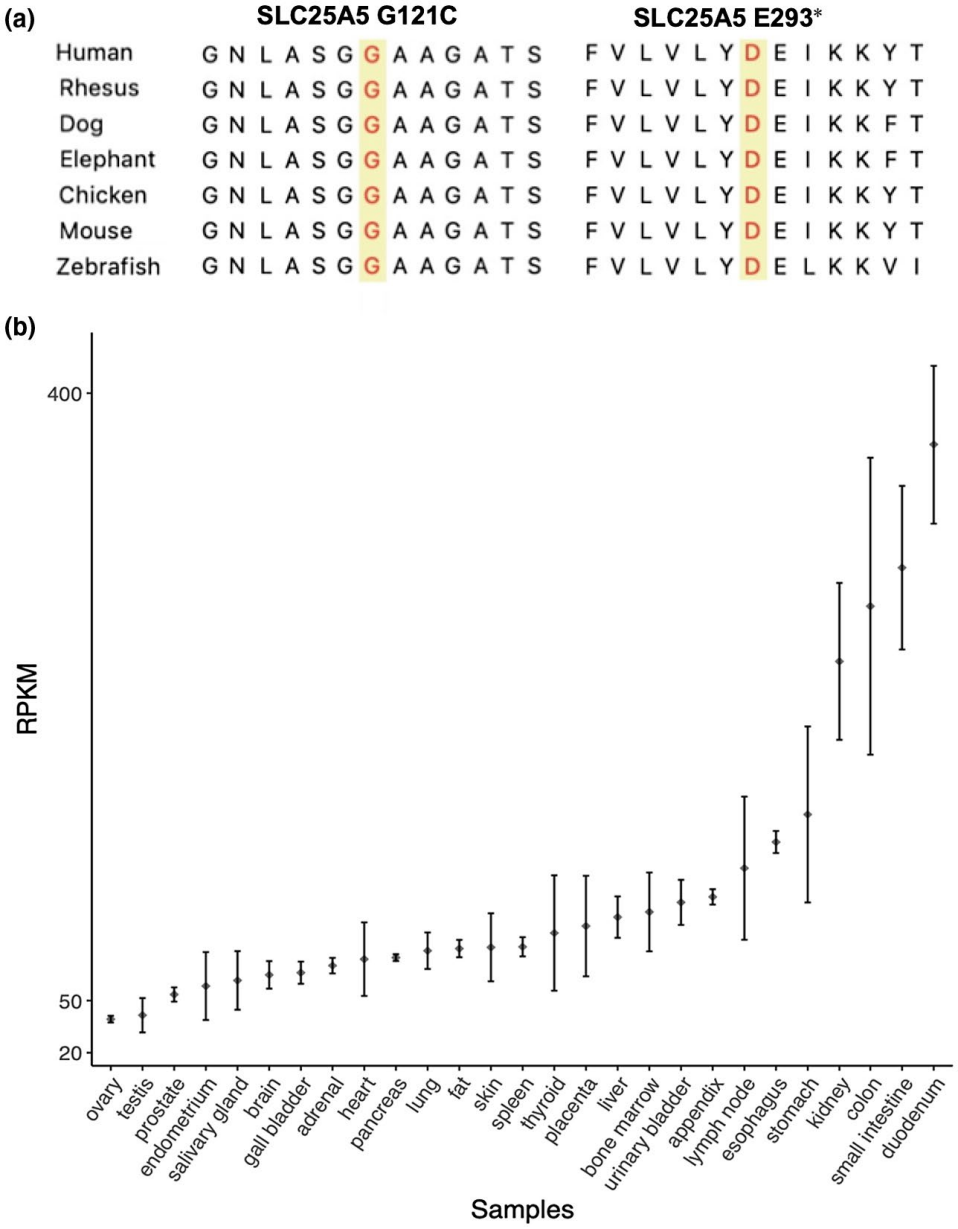
Genotypic and expression analysis of *SLC25A5*. (a) Conservation of SLC25A5 ortholog protein sequences across species. The mutant of interest is highlighted in yellow. (b) *SLC25A5* is differentially expressed in normal tissues based on NCBI annotation

In addition, two candidate loci were identified in chromosome X in our recruited family. These two rare damaging variants [p.Glu293* or p.E293* (NM_001152.4: c.877G>T) and p.Gly121Cys or p.G121C (NM_001152.4: c.361G>T)] were located in *SLC25A5* (OMIM#: 300150) (Table 1). All these variants did not exist in 1KGP. The p.Gly121Cys mutation of *SLC25A5* gene was found in the ExAC database with MAF < 0.01, while p.Glu293* in *SLC25A5* was a novel LoF variant and not found in the ExAC database. The SLC25A5 121 glycine residue and 293 glutamic residue are located in a highly conserved region across species (Figure 4a). All these variants were identified in all patients and is likely responsible for recessive inheritance. Furthermore, *SLC25A5* is expressed in testis tissue from NCBI browser (Figure 4b).

## 4 DISCUSSION

*AR* plays a pivotal role in the development of hypospadias. However, the research studies on dysregulated expression of *AR* are controversial and the exact mechanism is unclear. In the present study, we investigated the possible mechanisms leading to aberrant expression of genes involved in androgen metabolism in hypospadias by gene expression profiling and WES. Using data from gene expression profiling, we demonstrated that the significantly expressed genes are enriched in biological process of proteins interacting with ESR1. Additionally, we documented that mild and severe hypospadias express lower average transcript levels for *AR* and *CYP19A1*, whereas the expression of several steroidogenic enzymes, including *CYP3A4, HSD17B14*, *HSD3B7*, *HSD17B7*, and *CYP11A1* are significantly decreased in severe hypospadias, compared with non‐hypospadiac tissues. The genetic data further showed that the compound damaging variants of *AR* genes and other risk genes existed in severe hypospadias with the variable phenotypes.

Our findings suggest that AR and ESR1 signaling play a critical role in the development of mild and severe hypospadias using the gene expression profiling. The human *AR* gene, also known as the dihydrotestosterone receptor, is located on X‐chromosome and a member of the nuclear hormone receptor family. Although *AR* defect may play a causative role during the development of hypospadias, the *AR* expression (Balaji et al., 2020; Celayir et al., 2019; Lin et al., 2016; Pichler et al., 2013; Silva et al., 2013; Tack et al., 2019) in hypospadias is still debatable. Lin et al. (2016) and Silva et al. (2013) reported the decreased *AR* expression in boys with hypospadias compared with controls. Our study in Chinese children with hypospadias demonstrated the similar results to those studies. The finding of the aberrant expression of *AR* and other enzymes (*CYP19A1*, *CYP3A4, HSD17B14*, *HSD3B7*, *HSD17B7*, and *CYP11A1*), suggesting that the hypospadiac androgen synthesis may occur at various levels within individual patient. In addition, our findings suggests that genes encode for proteins interacting with ESR1 are enriched in hypospadias, indicating that ESR1 signaling plays an crucial role in prenatal penile development. The expression of estrogen receptor α in mouse fetal penile tissue was demonstrated in some previous studies, and women exposed to diethylstilbestrol in utero increased the risk of hypospadias (Kim et al., 2004; Klip et al., 2002). Our study provided preliminary evidence that the imbalance of AR and ESR1 signaling are present in hypospadias, which may affect the individual patient by various routes.

Furthermore, our genetic results identified a rare damaging *AR* allele (p.Arg841His) and other hypospadias risk mutants in all three hypospadias patients suggesting that compound variants might contribute to the genetic etiology of hypospadias in this family. The *AR* p.Arg841His variant is located in the ligand‐binding domain and has been previously reported in Asian patients with androgen intensitivity syndrome (Wang, Gong, Wang, & Qin, 2017), which is known to be associated with hypospadias. The *HSD3B1* gene, a member of *HSD3B* gene family, encodes the type I enzyme and is exclusively expressed in the adrenal, small intestine and placenta. *HSD3B1* expression is crucial to produce progesterone for the establishment and maintenance of pregnancy (Lai et al., 2017). Genetic mutation in *HSD3B1* is identified to be associated with prostate cancer susceptibility (Chang et al., 2002; Hearn et al., 2018). Carmichael and colleagues detected one SNP in *HSD3B1* which was associated with moderate hypospadias through sex hormone biosynthesis and metabolism (Carmichael, Witte, Ma, Lammer, & Shaw, 2014). In this family, a rare damaging variant p.Thr353Met in *HSD3B1* has been identified. Collectively, our study provided strong direct evidence that rare damaging variant p.Thr353Met in *HSD3B1* could increase hypospadias risk via sex hormone biosynthesis and metabolism. In addition to *AR* and *HSD3B1*, two rare damaging alleles in one novel candidate genes *SLC25A5* were identified in our recruited family. *SLC25A5* gene, previously named adenine nucleotide translocator 2 (*ANT2*), is ubiquitously expressed and is a transcriptionally active ADP/ATP translocase gene in human (Chen et al., 1990). *SLC25A5* has been related to non‐syndromic intellectual disability (Vandewalle et al., 2013). And the deletion encompassing *SLC25A43*, *LOC100303728*, *SLC25A5*, *CXorf56*, *UBE2A*, *NKRF*, and *SEPT6*, is associated with hypospadias (de Leeuw et al., 2010). However, the function of *SLC25A5* remains poorly understood. The data herein provided direct evidence that *SLC25A5* may contribute the genetic etiology of hypospadias.

Although genetic research in *AR* has been investigated for association with the risk of hypospadias (van der Zanden et al., 2012), the genetic contribution of *AR* gene to the risk of hypospadias is not clear. Rare damaging variants have a greater impact on protein function and have larger effects than common variants, which were used in identifying causal genes in birth defect disease (Chen, Kuang, Finnell, & Wang, 2018; Chen, Lei, Cao, et al., 2018; Chen, Lei, Zheng, et al., 2018; Lei et al., 2019). The diverse compound rare damaging variants of *AR* and other hypospadias risk mutants in hypospadias might be the major reason to cause the high interpatient variability. The AR network (Chen et al., 2019) indicated that ESR1 interacts with AR, and rare damaging variant (p.Thr353Met) in *HSD3B1* may also triggers a feedback loop, affecting the expression of the *AR*.

In conclusion, our study demonstrated that disrupted *AR* and *CYP19A1* expression could play a causative role in the development of hypospadias. Inconsistent *AR* expression may be caused by the regulation of ESR1 signaling or combined genetic effects of *AR* with other risk genes.

## CONFLICT OF INTEREST

The authors have declared that no competing interests exist.

## AUTHORS’ CONTRIBUTIONS

ZC, HX and FC directed and designed the study. ZC, XL and YW contributed to data collection. ZC and XL wrote the paper. ZC, HX and FC supervised the work. All authors read and approved the final manuscript.

## Supporting information


**Table S1**

## Data Availability

They are available on special request.
