# Supplementary material for: Dysregulated expression of androgen metabolism genes and genetic analysis in hypospadias
Source: Mol Genet Genomic Med. 2020 Jun 8;8(8):e1346. doi: 10.1002/mgg3.1346 (PMC7434757; doi:10.1002/mgg3.1346)
Supplement: Supplementary file 1 — Table S1 [file MGG3-8-e1346-s001.docx]

**Table S1.** Student’s t-test p-values were used to measure differential gene expression related to androgen metabolism between hypospadiac foreskins and non-hypospadiac forskins.

| Gene symbol | Ensembl gene | Gene description | *P*_severe_^a^ | *P*_mild_^b^ |
| --- | --- | --- | --- | --- |
| *CYP19A1* | ENSG00000137869 | Cytochrome P450 family 19 subfamily A member 1 | 4.8×10^-3^ | 1.8×10^-2^ |
| *AR* | ENSG00000169083 | Androgen receptor | 5.0×10^-3^ | 3.9×10^-2^ |
| *CYP3A4* | ENSG00000160868 | Cytochrome P450 family 3 subfamily A member 4 | 2.1×10^-2^ | 0.72 |
| *HSD17B14* | ENSG00000087076 | Hydroxysteroid 17-beta dehydrogenase 14 | 2.5×10^-2^ | 0.28 |
| *HSD3B7* | ENSG00000099377 | Hydroxy-delta-5-steroid dehydrogenase, 3 beta- and steroid delta-isomerase 7 | 3.0×10^-2^ | 0.10 |
| *HSD17B7* | ENSG00000132196 | Hydroxysteroid 17-beta dehydrogenase 7 | 4.2×10^-2^ | 0.91 |
| *CYP11A1* | ENSG00000140459 | Cytochrome P450 family 11 subfamily A member 1 | 4.8×10^-2^ | 0.13 |
| *HSD17B3* | ENSG00000130948 | Hydroxysteroid 17-beta dehydrogenase 3 | 0.06 | 0.17 |
| *HSD3B1* | ENSG00000203857 | Hydroxy-delta-5-steroid dehydrogenase, 3 beta- and steroid delta-isomerase 1 | 0.08 | 0.26 |
| *CYP21A2* | ENSG00000231852 | Cytochrome P450 family 21 subfamily A member 2 | 0.11 | 0.24 |
| *HSD17B13* | ENSG00000170509 | Hydroxysteroid 17-beta dehydrogenase 13 | 0.13 | 0.90 |
| *SRD5A3* | ENSG00000128039 | Steroid 5 alpha-reductase 3 | 0.14 | 0.89 |
| *HSD3B2* | ENSG00000203859 | Hydroxy-delta-5-steroid dehydrogenase, 3 beta- and steroid delta-isomerase 2 | 0.15 | 0.19 |
| *AKR1C2* | ENSG00000151632 | Aldo-keto reductase family 1 member C2 | 0.16 | 0.31 |
| *CYP3A7* | ENSG00000160870 | Cytochrome P450 family 3 subfamily A member 7 | 0.166 | 0.46 |
| *HSD17B2* | ENSG00000086696 | Hydroxysteroid 17-beta dehydrogenase 2 | 0.19 | 0.439 |
| *HSD17B1* | ENSG00000108786 | Hydroxysteroid 17-beta dehydrogenase 1 | 0.22 | 0.69 |
| *HSD17B6* | ENSG00000025423 | Hydroxysteroid 17-beta dehydrogenase 6 | 0.22 | 0.28 |
| *STAR* | ENSG00000147465 | Steroidogenic acute regulatory protein | 0.30 | 0.07 |
| *AKR1C1* | ENSG00000187134 | Aldo-keto reductase family 1 member C1 | 0.32 | 0.81 |
| *AKR1C3* | ENSG00000196139 | Aldo-keto reductase family 1 member C3 | 0.35 | 0.72 |
| *HSD17B11* | ENSG00000198189 | Hydroxysteroid 17-beta dehydrogenase 11 | 0.40 | 0.92 |
| *AKR1C4* | ENSG00000198610 | Aldo-keto reductase family 1 member C4 | 0.44 | 0.16 |
| *CYB5A* | ENSG00000166347 | Cytochrome b5 type A | 0.46 | 0.06 |
| *HSD17B10* | ENSG00000072506 | Hydroxysteroid 17-beta dehydrogenase 10 | 0.47 | 0.45 |
| *SRD5A1* | ENSG00000145545 | Steroid 5 alpha-reductase 1 | 0.60 | 0.23 |
| *HSD17B8* | ENSG00000204228 | Hydroxysteroid 17-beta dehydrogenase 8 | 0.67 | 0.95 |
| *SRD5A2* | ENSG00000277893 | Steroid 5 alpha-reductase 2 | 0.70 | 0.66 |
| *HSD17B4* | ENSG00000133835 | Hydroxysteroid 17-beta dehydrogenase 4 | 0.71 | 0.47 |
| *RDH5* | ENSG00000135437 | Retinol dehydrogenase 5 | 0.71 | 0.73 |
| *SHBG* | ENSG00000129214 | Sex hormone binding globulin | 0.75 | 0.39 |
| *CYP17A1* | ENSG00000148795 | Cytochrome P450 family 17 subfamily A member 1 | 0.76 | 0.61 |
| *CYP3A5* | ENSG00000106258 | Cytochrome P450 family 3 subfamily A member 5 | 0.90 | 0.81 |
| *HSD17B12* | ENSG00000149084 | Hydroxysteroid 17-beta dehydrogenase 12 | 0.96 | 0.40 |

^a^Student’s t-test p-values between severe hypospadiac foreskins and normal forskins.

^a^Student’s t-test p-values between mild hypospadiac foreskins and normal forskins.
